# Illustrated instructions for mechanical quality assurance of a medical linear accelerator

**DOI:** 10.1002/acm2.12265

**Published:** 2018-03-03

**Authors:** Laurence Court, He Wang, David Aten, Derek Brown, Hannelie MacGregor, Monique du Toit, Melinda Chi, Song Gao, Adam Yock, Michalis Aristophanous, Peter Balter

**Affiliations:** ^1^ The University of Texas MD Anderson Cancer Center Houston TX USA; ^2^ University of California San Diego CA USA; ^3^ Department of Radiation Oncology Groote Schuur Hospital University of Cape Town Cape Town South Africa; ^4^ Stellenbosch University and Tygerberg Hospital Cape Town South Africa; ^5^ Vanderbilt University Medical Center Nashville TN USA

**Keywords:** quality assurance, training

## Abstract

**Purpose:**

The purpose of this study was to develop and test a set of illustrated instructions for effective training for mechanical quality assurance (QA) of medical linear accelerators (linac).

**Methods:**

Illustrated instructions were created for mechanical QA and underwent several steps of review, testing, and refinement. Eleven testers with no recent QA experience were then recruited from our radiotherapy department (one student, two computational scientists, and eight dosimetrists). This group was selected because they have experience of radiation therapy but no preconceived ideas about how to do QA. The following parameters were progressively decalibrated on a Varian C‐series linac: Group A = gantry angle, ceiling laser position, X1 jaw position, couch longitudinal position, physical graticule position (five testers); Group B = Group A + wall laser position, couch lateral and vertical position, collimator angle (three testers); Group C = Group B + couch angle, wall laser angle, and optical distance indicator (three testers). Testers were taught how to use the linac and then used the instructions to try to identify these errors. An experienced physicist observed each session, giving support on machine operation as necessary.

**Results:**

Testers were able to follow the instructions. They determined gantry, collimator, and couch angle errors within 0.4°, 0.3°, and 0.9° of the actual changed values, respectively. Laser positions were determined within 1 mm and jaw positions within 2 mm. Couch position errors were determined within 2 mm and 3 mm for lateral/longitudinal and vertical errors, respectively. Accessory‐positioning errors were determined within 1 mm. Optical distance indicator errors were determined within 2 mm when comparing with distance sticks and 6 mm when using blocks, indicating that distance sticks should be the preferred approach for inexperienced staff.

**Conclusions:**

Inexperienced users were able to follow these instructions and catch errors within the criteria suggested by AAPM TG‐142 for linacs used for intensity‐modulated radiation therapy. These instructions are, therefore, suitable for QA training.

## 1 INTRODUCTION

The goal of linear accelerator (linac) quality assurance (QA) is to ensure that the device does not significantly deviate from its baseline values, with the underlying principle that the dose delivered to the patient should be within 5% of the prescribed dose.^1^, ^2^, ^3^ There are many guidance documents indicating appropriate QA criteria,^1^, ^3^, ^4^, ^5^, ^6^ some of which include some discussion on the experimental techniques. There are, however, two aspects of QA that are sometimes neglected. The first is the creation of clear documents that fully describe the procedures to test the treatment device. This is important for effective training and also for inexperienced users who need a reminder on specific aspects of QA procedures. The second aspect of QA that requires more exploration is the testing of procedure documents to ensure that users can follow the procedures and actually catch errors. In this report, we describe the creation of clear instructions for the tests needed for mechanical QA of medical accelerators, including full fault insertion testing. That is, the intentional insertion of errors to determine whether users can catch them. To maximize the usefulness of the instruction set, including its potential utility for training of physicists who are not native English speakers, the decision was made to focus on good illustrations to clarify the written instructions.

## 2 MATERIALS AND METHODS

The basis of this report is written instructions created to act as a reminder for junior medical physicists when performing monthly mechanical QA. These instructions were first edited for simplicity. Although combining multiple tests into a single test can improve efficiency, in these instructions we generally described tests one at a time to maintain clarity. Once the instructions for these tests were prepared, they were then discussed and edited by several groups of physicists (total 12 physicists) with extensive experience in mechanical QA. A professional illustrator then created the illustrations, with input from an experienced physicist. The first version of the instructions was then used by three medical physics graduate students with no experience in mechanical QA, observed by an experienced physicist. The students were asked to run through all of the tests. Unclear parts of the instructions and illustrations were edited, and the instructions were then reviewed by professional editorial staff to simplify the English language.

Testers with no recent QA experience were recruited from our radiotherapy department (one student, two computational scientists, and eight dosimetrists). Each of these testers used the instructions to perform QA testing of a nonclinical medical accelerator (Varian 2100 C/D, Millenium MLC). Each tester performed the tests alone, observed only by an experienced physicist. The testing started with an explanation of the goals of the study and a brief explanation of how to use the hand pendant (5–10 min). The tester then performed the QA testing with no additional input from the physicist unless they required additional instructions on how to move the linac (e.g., selecting independent jaws on the hand pendant). No assistance was offered to understand the instructions. Sixteen tests (see Appendix S1) were evaluated, although some tests were shortened to reduce the testing time. Specifically, test 11 (jaw readouts vs light field) was performed only for the X1 jaw, test 12 (multileaf collimator [MLC] position vs light field) was omitted, and test 13a (physical wedge position check) was performed only for a 60° physical wedge.

Prior to the testing, several linac parameters were intentionally decalibrated. This decalibration was carried out in sets (Table 1) with each subsequent set of decalibrations adding to that already in place. Testers were told that some parameters would not pass the tests but were not given any other guidance. The numbers of testers for Group A, Group B, and Group C were five, three, and three, respectively. For each QA test, the maximum difference between the value measured by any single tester and an experienced physicist was noted as an estimation of the accuracy of the test when performed by an inexperienced user. Based on the experience of this extensive testing, together with additional feedback from physicists at other institutions in the USA and South Africa, small edits were made to clarify some of the text, and a test that was difficult for the testers to understand (and reliably perform) was replaced. This is discussed further in the Results section. Finally, additional edits were made based on input from the reviewers of this manuscript.

**Table 1:** Groups of linac parameters that were decalibrated for the fault insertion testing. Decalibrations were additive, so Group B includes all of the Group A changes, and Group C includes all of Group A and B changes

Group A (5 testers)	Group B (3 testers)	Group C (3 testers)
Gantry angle 0.0° → 358.8 90.0° → 89.0 180.0° → 178.8 270.0° → 268.8 Ceiling laser position Lateral shift 2 mm X1 jaw position 0.0 cm → 0.5 cm 10.0 cm → 10.3 cm 20.0 cm → 20.3 cm Couch longitudinal position 140.0 cm → 140.7 cm Physical graticule position superior direction 1.5 mm	Wall laser position left laser shifted superior 3 mm Couch lateral position 0.0 cm → 999.6 cm Couch vertical position 0.0 cm → 0.4 cm Collimator angle 0.0° → 0.4 90.0° → 88.6	Couch angle 0.0° → 1.7° 90.0° → 94.7° 270.0° → 269.6° Wall laser angle Left laser orientation 4 mm (measured at exit window of linac) Optical distance indicator 100.0 cm → 100.8 cm 110.0 cm → 109.5 cm

## 3 RESULTS

The final illustrated instructions for mechanical QA are reproduced in Appendix S1.

A list of necessary equipment and a section to clarify the terminology used throughout the instructions were added to the instructions based on initial feedback. Furthermore, based on the initial experience, clearer illustrations of what the user should be looking for or comparing were added (e.g., the figure after task 5 of test 9).

The maximum discrepancy between the testers’ measurements and the actual values determined by an experienced physicist are shown in Table 2, which also includes the pass/fail criteria suggested in TG‐142. In all tests except one, the greatest disagreement between the testers and the experienced physicist was still within the TG‐142 criteria. The one exception to this was the use of a vertical surface to check the optical distance indicator (ODI), where our inexperienced testers had a maximum error of 6 mm (test 6b). Based on this experience, this test was replaced with a test that uses the mechanical distance indicator (test 6a).

**Table 2:** Maximum discrepancy between the testers’ measurements and the experienced physicist's measurements (i.e., ground truth). Test numbers refer to the tests in the instructions (Appendix S1). Test 6b is measuring the same as test 6, but with a different approach. Based on these results, test 6b was abandoned for inexperienced users. The TG‐142 criteria are from Table 2 (monthly) of that report unless noted otherwise

Test	Parameter	Criteria from TG‐142 (IMRT machine)	Maximum discrepancy
Test 1	Gantry angle vs readout	1.0°	0.4°
Test 2	Collimator angle vs readout	1.0°	0.3°
Test 3	Left wall laser orientation	–	2 mm
Test 4	Right wall laser alignment (horizontal laser)	1 mm	1 mm
Test 5	ODI @ 100 cm	2 mm (TG‐142, table I, daily)	2 mm
Test 6a	ODI (other distances, relative to 100 cm point)	2 mm (TG‐142, table I, daily)	2 mm
Test 6b*	ODI (other distances, relative to 100 cm point)	2 mm (TG‐142, table I, daily)	6 mm
Test 7	Ceiling laser alignment	1 mm	1 mm
Test 8	Ceiling laser orientation	na	Not tested
Test 9	Left wall laser alignment (vertical laser)	1 mm	1 mm
Test 10	Crosshair centering	1 mm	Not tested
Test 11	Jaw readouts vs light field (Y1 jaw, asymmetric)	1 mm	1 mm
Test 12	MLC pattern check (using light field)	–	Not tested
Test 13a	Physical wedge position check	2 mm	1 mm
Test 13b	Physical graticule position	2 mm	1 mm
Test 14	Couch angle vs readout	1.0°	0.9°
	Couch centering	–	Not tested
Test 15a	Couch relative positions	–	Not tested
Test 16	Couch absolute position (isocenter)	2 mm	2 mm (lateral and longitudinal)
			3 mm (vertical)
Test 17	Accessory position check		1 mm
Test 18	Safety tests		Not tested

ODI, optical distance indicator; MLC, multileaf collimator.

## 4 DISCUSSION

We have created illustrated instructions that can be employed to guide the procedures used for the mechanical QA of medical linacs. We tested these instructions to ensure that they can be used effectively by inexperienced users to catch errors in the mechanical settings of the machine. We also incorporated helpful feedback from physicists at multiple institutions. In almost all cases, inexperienced users were able to catch errors within the criteria suggested in TG‐142 (intensity‐modulated radiation therapy [IMRT] machine). This indicates that the procedures are appropriate and the instructions sufficiently clear that they can support the training new staff who will be responsible for mechanical QA. The one exception was the couch vertical position (test 16), for which the maximum discrepancy was 3 mm. This was only for one tester, with all other testers’ results falling at 2 mm or less. The reasons for the discrepancy in this one tester's result are unclear, although this, perhaps, indicates a limitation of fault testing: many testers are needed to fully understand the tools, so our data can really be considered only as representative and not proof that users will always find errors.

Although the testers successfully completed all tests, this was not always a smooth process. For example, some testers had a tendency to skip steps or complete tasks. We found that, when testers did this, they quickly got confused and had to go back. One example is the task of setting the graph paper on the treatment couch (preparation task immediately before test 7). Several testers initially unintentionally omitted this task completely, but soon realized that they could not continue without graph paper and went back to find the task that they had initially omitted. In no case, a tester skipped a step and did not go back to it. In response to this experience, we added text to the graph paper preparation task noting that this task must be completed before starting the next set of tests.

Since the creation of these instructions, we have used them for training graduate medical physics students at one of our institutions (>20 students). In one version of that training, we decalibrated an old mechanical distance stick and asked the students to find what had been decalibrated (we implied that this was the linac, but we were unable to actually decalibrate the linac itself as it was in clinical use). The students (operating in groups of about four), who had no experience in mechanical QA, were able to identify the problem with minimal feedback from the instructors. These instructions were also employed for training at a second location, with positive feedback.

One limitation of this study is the fact that not all induced errors were larger than the TG‐142 tolerances, meaning that, even after the decalibration, the parameter would have passed the TG‐142 tolerance. Furthermore, in most cases, only one decalibration was performed for each linac parameter (exceptions are parameters such as gantry angle, which are tested at multiple points). This limitation was necessary because of the time required to test each scenario, and we believe that the final results are still reasonable estimates of the mechanical QA accuracy possible with these procedures. A second limitation is that these instructions were developed and tested using Varian linacs. Although these instructions have been used with Elekta treatment devices, the testing with non‐Varian linacs has not been as thorough as that with Varian linacs. Users of such machines may have to make some changes to the instructions. This is also the case for users of other treatment devices.

We did not investigate the impact of measurement uncertainties on false‐positive or false‐negative rates for QA. That is, we did not investigate the frequency with which users might identify a failure that is not true or pass a test that should have failed. We, however, compared the measurement variation with typical QA criteria. Although there will certainly be incorrect measurements, the reasonably small measurement variation implies that the clinical impact of these should be small.

Many medical physicists throughout the world work on their own. Although they are often able to attend training, this can be offsite and may be held sometime before their treatment device is actually installed. Based on the results of the work presented here, we believe that these instructions could form a useful basis for these physicists in achieving an effective mechanical QA procedure for their machines. Given the success with which the inexperienced testers managed to catch our intentional errors, these results also indicate that, in the absence of sufficient qualified staff, some of these tasks could be delegated to less experienced staff (with appropriate supervision, of course). These instructions do not, however, offer any guidance on diagnosing any errors that are found. It is important to note that the interdependence of various parameters being tested here means that the cause of the failure is not necessarily obvious, and well‐trained staff are still needed to determine the cause of any failures.

Our experience is that the creation of these instructions required multiple iterations, and the challenge of creating simple, easy‐to‐follow instructions that can reliably catch errors is extremely difficult and time‐consuming. We also found that use testing is extremely important (and time‐consuming). The final result, however, is a thoroughly tested set of instructions. The development of such instructions for a wider range of tests should facilitate training of the next generation of QA experts.

## 5 CONCLUSIONS

We created and tested illustrated instructions for performing mechanical QA of a medical linac. With these instructions, inexperienced testers were able to catch intentional errors with an accuracy within the range of the pass/fail criteria suggested in TG‐142.

## CONFLICT OF INTEREST

The authors report no conflicts of interest.

## Supporting information


**Appendix S1.** Instructions for mechanical QA.
