# Supplementary material for: Illustrated instructions for mechanical quality assurance of a medical linear accelerator
Source: J Appl Clin Med Phys. 2018 Mar 3;19(3):355–9. doi: 10.1002/acm2.12265 (PMC5978554; doi:10.1002/acm2.12265)
Supplement: Supplementary file 1 — Appendix S1. Instructions for mechanical QA. [file ACM2-19-355-s001.pdf]

Illustrated instructions for mechanical Quality Assurance of a medical linear accelerator

Appendix S: Instructions for mechanical QA

Laurence Court[1], He Wang[1], Dave Aten[1], Derek Brown[2], Hannelie MacGregor[3], Monique du Toit[4], Melinda Chi[1], Song Gao[1], Adam Yock[5], Michalis Aristophanous[1], Peter Balter[1]

(1) The University of Texas MD Anderson Cancer Center, Houston, TX, USA

(2) University of California, San Diego, CA, USA

(3) Department of Radiation Oncology, Groote Schuur Hospital and University of Cape Town,

(4) Cape Town, South Africa

(5) Stellenbosch University and Tygerberg Hospital, Cape Town, South Africa

(6) Vanderbilt University Medical Center, Nashville, TN, USA

Corresponding author:

Laurence Court

Department of Radiation Physics

The University of Texas MD Anderson Cancer Center

1400 Pressler Street, Unit 1420

Houston TX 77030

Tel.: (713) 563 2546

Email: lecourt@mdanderson.org

Note: This is the online appendix for a manuscript in the Journal of Applied Clinical Medical Physics, 2018.

Appendix S: Instructions for mechanical QA
This document details the procedures for testing the mechanical accuracy/precision of the LINAC and associated equipment. It is suggested that the procedures be carried out in the order in which they are listed here. Square brackets, [], indicate a step that should already have happened. *Hints* (in italics) indicate practical hints, or changes in the test order that can be useful.

Users should verify and edit the tolerance values for each test according to their own equipment/environment.

Necessary equipment

- □ These instructions
- □ QA results sheet (at the end of these instructions)
- □ Blank paper
- □ Sticking tape

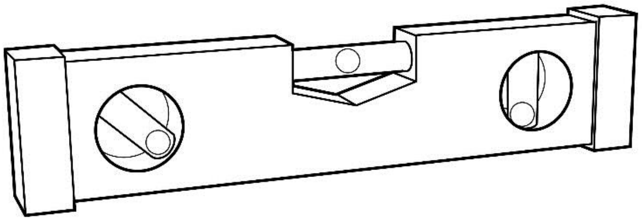

□ Level (Spirit level or digital level) (preferably with a magnetic base)

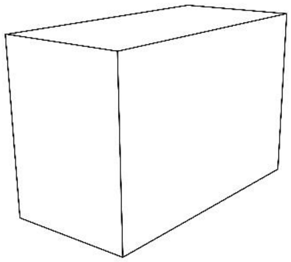

□ Block (with perpendicular edges) (minimum 20cm high, preferably 30cm high)

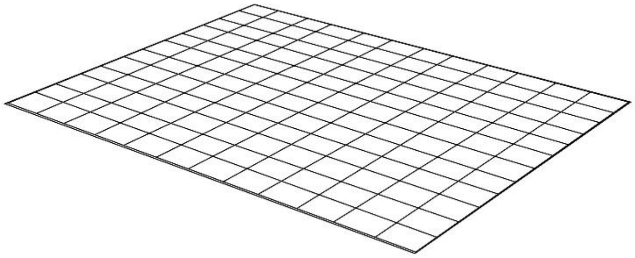

□ Graph paper

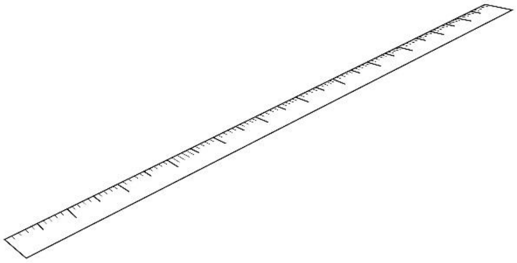

□ Accurate ruler

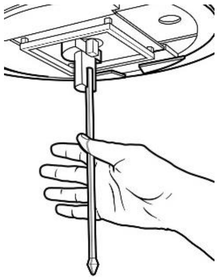

□ Mechanical Distance Indicator (and holder)

The treatment room

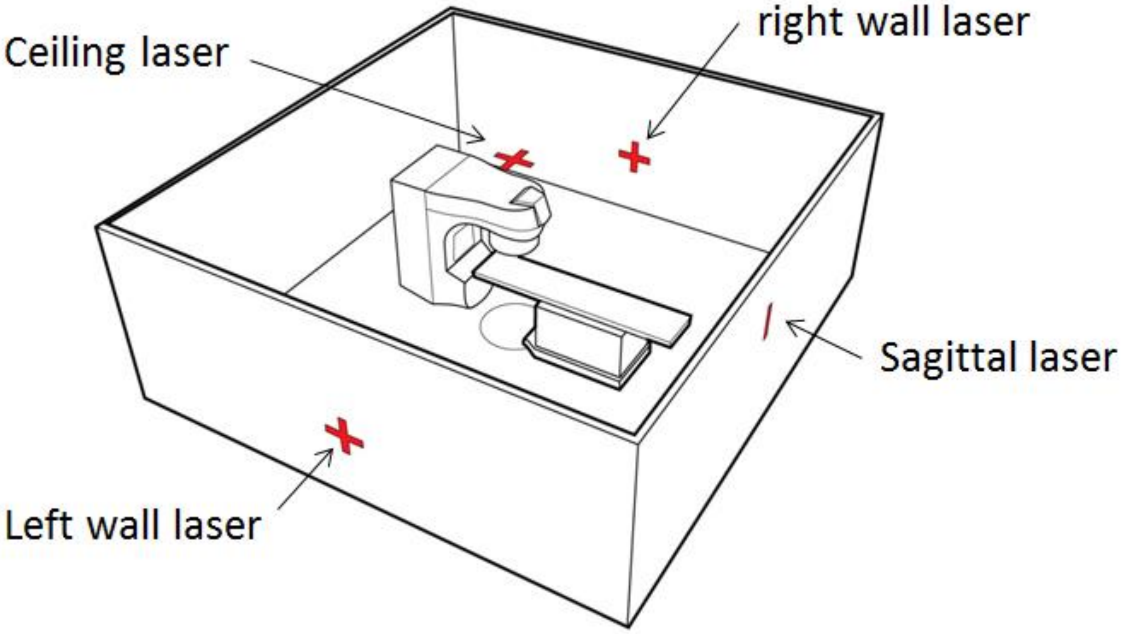

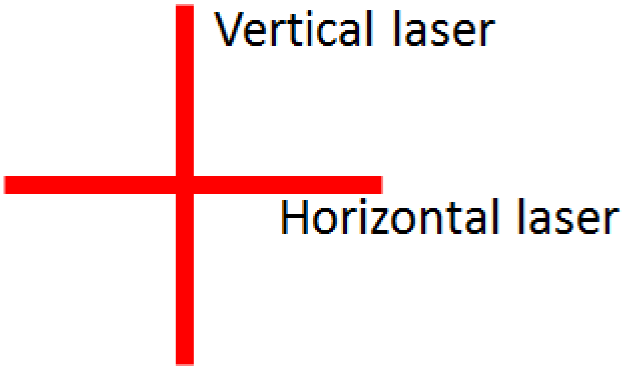

Terminology

Couch longitudinal:	in/out
Couch lateral:	left/right
Couch Vertical:	up/down

Test 1: Gantry angle vs. readouts

Purpose: To check the accuracy of the gantry readout with the actual gantry position

Tolerance: 1.0°

Procedure:

1. Place the Level (Spirit Level or Digital Level) on flat surface behind the exit window of the LINAC, in line with the direction of rotation, as shown in the figure.

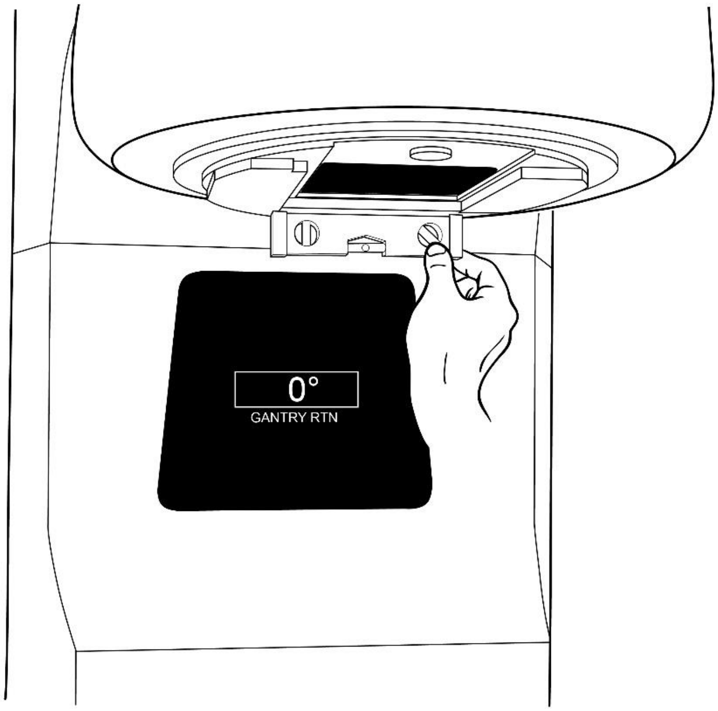

2. Rotate the gantry and collimator to approximately 0° using the digital readouts.
3. Carefully adjust the gantry angle until the Level shows it is level (0°), read the gantry angle on the digital readout of the linear accelerator, and record this value.

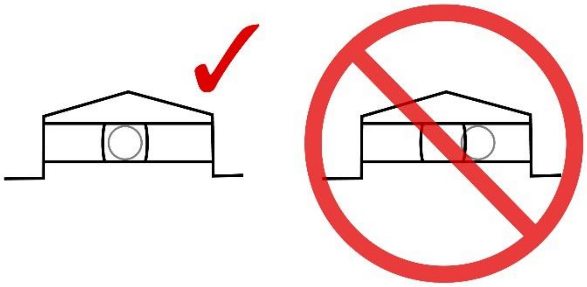

- **Note:** for 0° and 180° use bubble labeled A; for 90° and 270° use bubble labeled B.

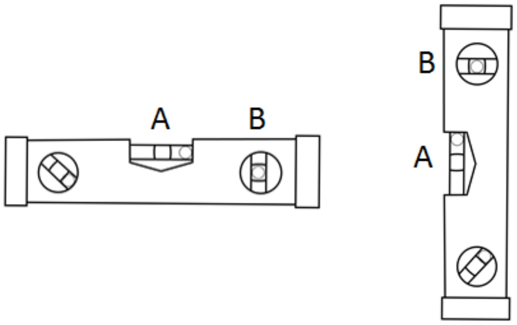

4. Rotate the gantry to 90° using the digital readouts.
5. Carefully adjust the gantry angle until the Level shows it is level (90°), read the gantry angle on the digital readout of the linear accelerator, and record this value.

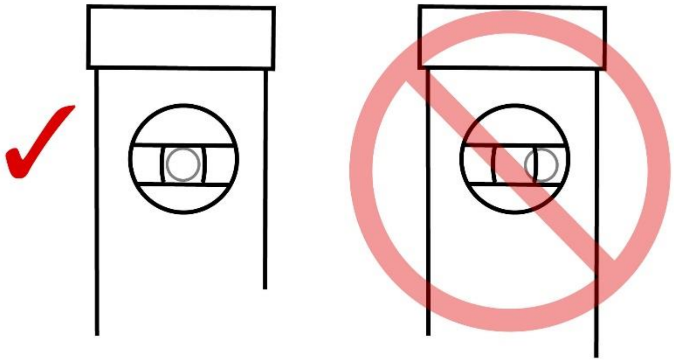

6. Repeat steps 4 and 5 for gantry angles of 180°, 270° and 180° - Note: 180° is measured in both directions.

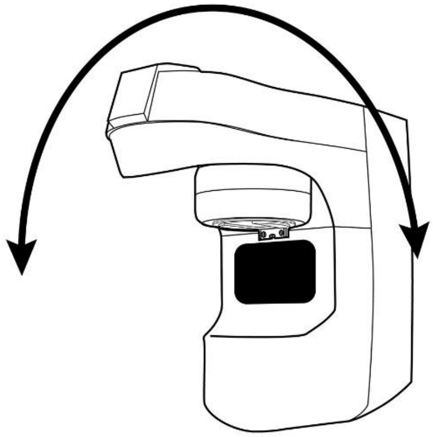

Note: Subsequent tests should use the exact (true) gantry positions, not the digital readouts.

Test 2: Collimator angle vs. readout
Purpose: To check the accuracy of the collimator angle readout with the actual collimator position
Tolerance: 1.0°
Procedure:

1. Set the gantry to exactly 90° using the Level (as in Test 1).

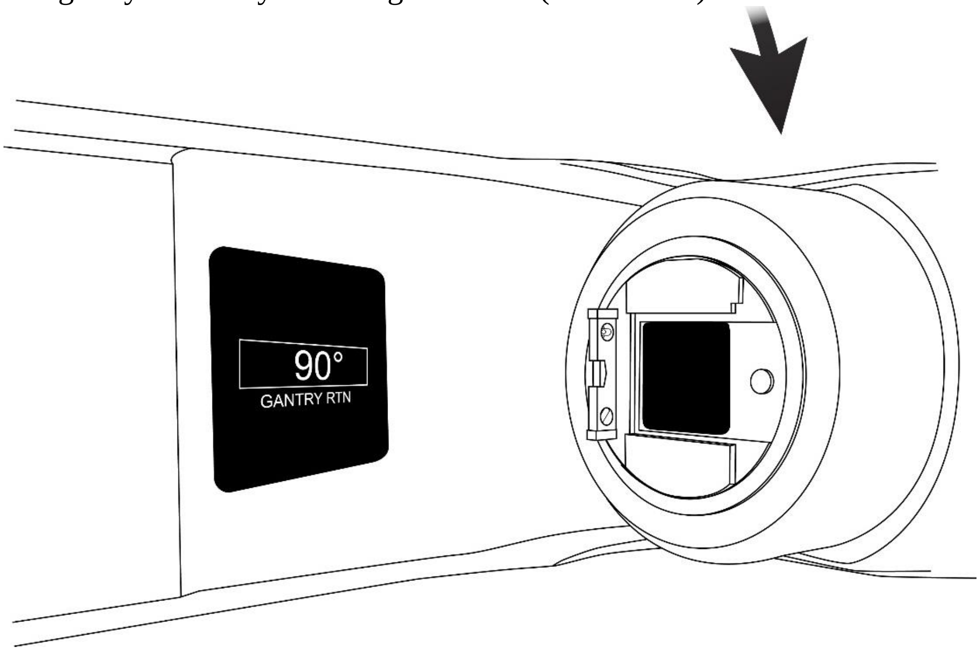

2. Move the Level to the Teflon edge where the blocks slide in (Varian linac) – see diagram below - and rotate the collimator to approximately 0°

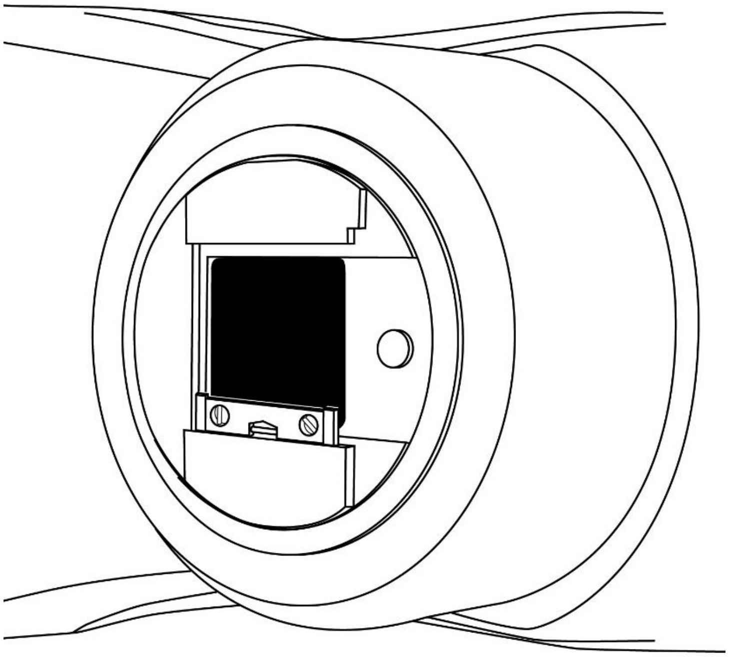

3. Carefully adjust the collimator angle until the Level shows that it is level (Bubble A). Read the collimator angle on the digital readout of the linear accelerator, and record this value.
4. Repeat steps 2 and 3 for collimator angles of 90° and 270° (Bubble B for 90° and 270°). At each position, carefully adjust the collimator angle using the Level. Read the collimator angle on the digital readout of the linear accelerator, and record this value.

Note: This test assumes that the Teflon is parallel to the jaw, and the user should first demonstrate that this is the case. One way to do this is move the jaw to a position close to the upper side of the Level (when in the position in the figure above), and to check whether the shadows of the light field and the level are parallel. Any differences need to be accounted for in subsequent tests.

Test 3: Test vertical and horizontal lasers orientations (the ‘wall lasers’)
Purpose: To check wall lasers are orientated correctly
Tolerance: 2 mm (at linac exit window)

1. [Rotate Gantry to exactly 90° using the Level, and collimator angle to 0° using the Level, as above]
2. Check that the left wall lasers are aligned with the crosshair on the exit window of the linac

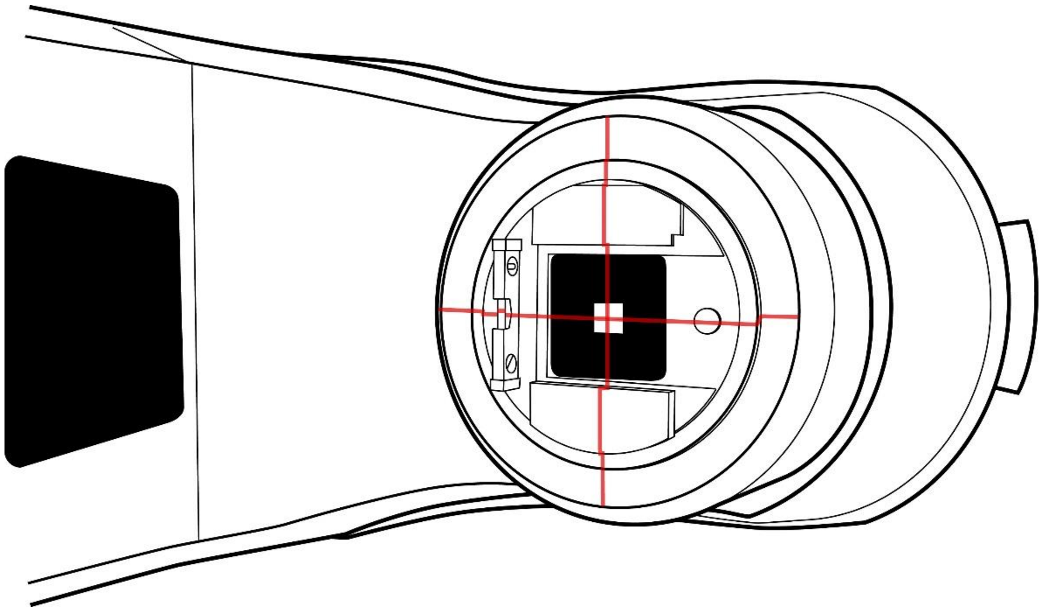

3. Record the distance between the left wall lasers and the crosshairs at the exit window of the linac

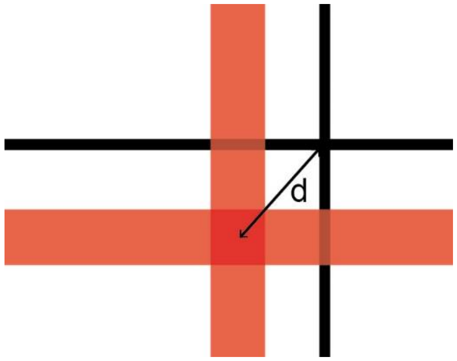

4. Rotate the gantry to exactly 270° (using the Level, as described in Test 1)

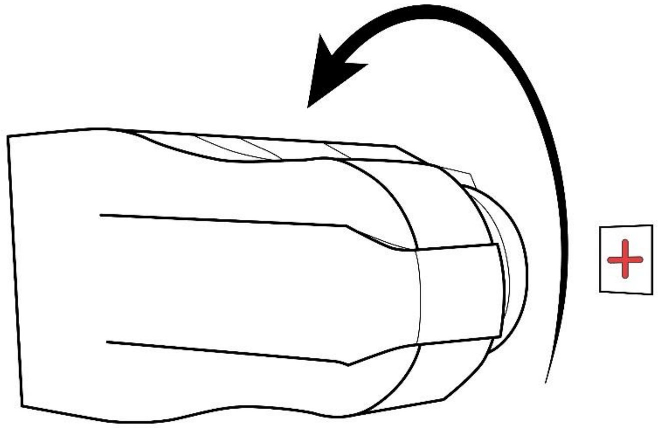

5. Record the distance between the right wall lasers and the crosshairs at the exit window of the linac

Note: Tests 4 and 9 are also important tests of the wall lasers. If the lasers fail <u>any</u> of these tests, the user must examine the lasers positioning and orientation carefully to evaluate the real cause of the failure. Also, step 8 of Test 11 checks that the crosshair is parallel to the jaws.

Test 4: Horizontal laser alignment ('horizontal wall laser')
Purpose: To check the agreement of the side (wall) lasers with isocenter (horizontal lasers only)
Tolerance: 2 mm

1. Rotate gantry to exactly 0° and collimator to exactly 0° *(hint: use the digital values recorded in tests 1 and 2)*
2. Insert 100cm mechanical distance indicator (MDI) (holder + stick), and align the 100cm mark on the stick to the bar on the holder.

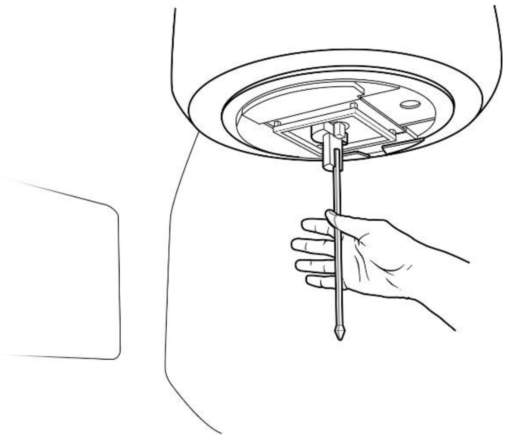

3. Hold paper up so the left wall lasers can be seen *(hint: if you use graph paper, it makes estimating errors easier)*

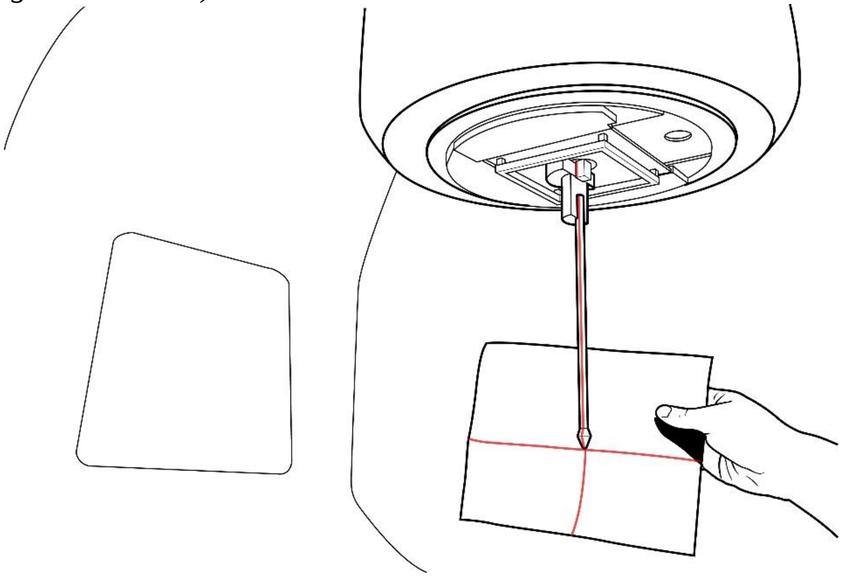

4. Record the distance from the MDI tip to the center of the left wall horizontal laser (ignore the position of the vertical laser)

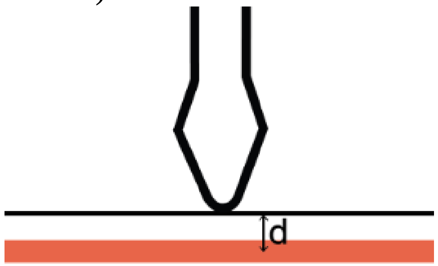

5. Repeat for the right wall horizontal laser. Record the distance from the MDI tip to the center of the right wall horizontal laser.

Test 5: Optical distance indicator (ODI) @100cm

Purpose: To test the accuracy of the ODI at 100cm

Tolerance: 2 mm

Procedure:

1. Set gantry and collimator to 0°.
2. Attach the 100cm Mechanical Distance Indicator (MDI) to the gantry
3. Slowly move the treatment couch vertically until it reaches the MDI.

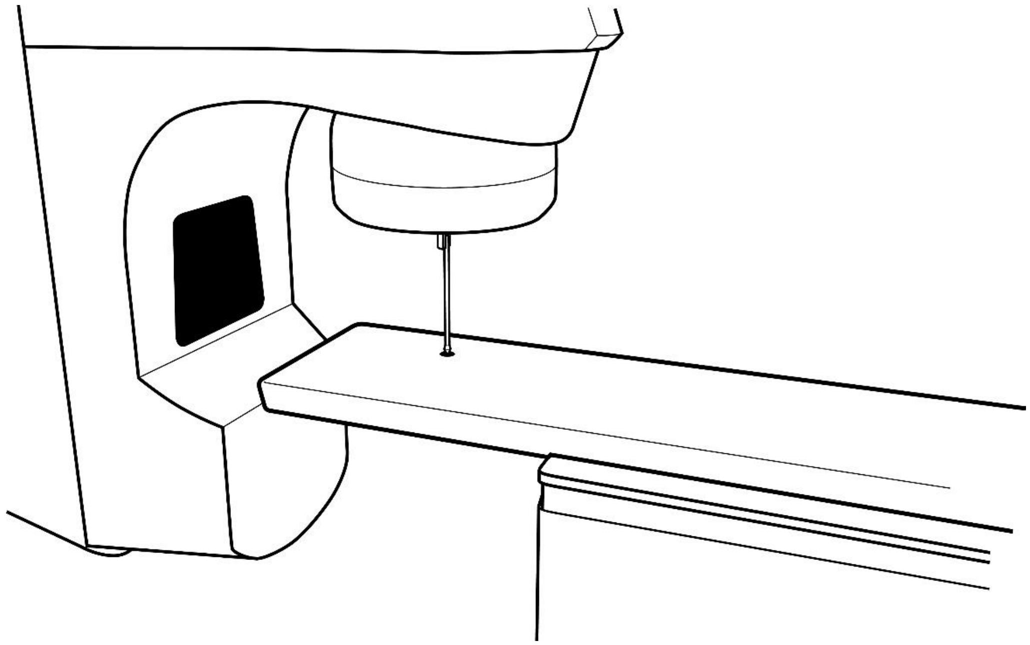

4. Remove the MDI (and holder), turn the ODI and field light on, and check that it reads 100cm at the crosshair. Placing paper on the couch can help see the ODI and crosshair.

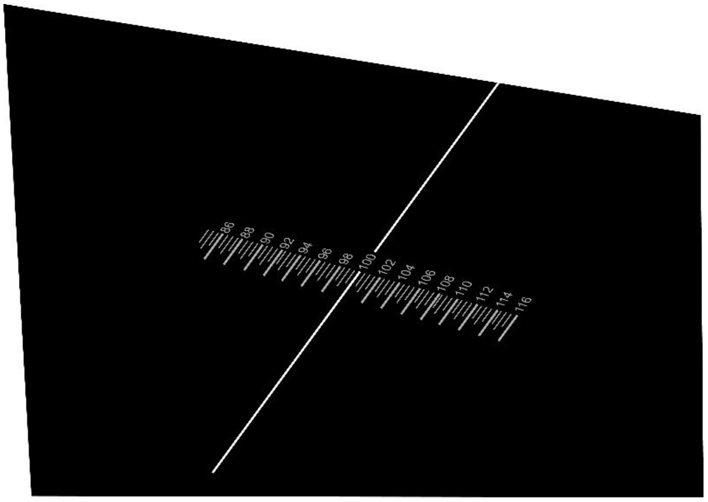

5. Record the error in the ODI reading.

- **Note**: if the ODI is off, do not rely on it for subsequent tests.

Test 6: Optical distance indicator (ODI) – range of distances
Purpose: To test the accuracy of the ODI at a range of distances (relative)
Tolerance: 2 mm

Two methods are described here. The first is easier, and it was found that inexperienced users had more accurate results using this method. The second is quicker, but can be more difficult to understand, and is sensitive to experimental setup – testing showed that inexperienced users had trouble with this test.

Test 6a: Optical distance indicator (ODI) – range of distances
Procedure:

1. Set gantry and collimator to 0°.
2. Attach the 80cm Mechanical Distance Indicator (MDI) to the gantry
3. Slowly move the treatment couch vertically until it reaches the MDI. *(hint: if your couch will not move this far, use blocks on the surface of the couch)*

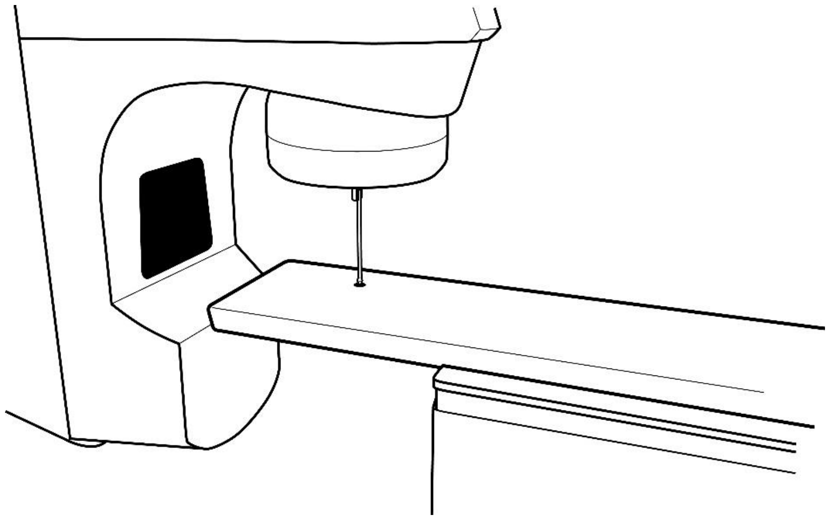

4. Remove the MDI (and holder), turn the ODI and field light on, and check that it reads 80cm at the crosshair. Placing paper on the couch can help see the ODI and crosshair. Record the error in the ODI reading.
5. Repeat this using the 90cm and 110cm Mechanical Distance indicators.

Test 6b: Optical distance indicator (ODI) – range of distances
This is an alternative to Test 6a. It requires more experience, and testing showed that it can be less reliable than Test 6.

Procedure:

1. Set gantry and collimator to 0°, set the couch to approximately 10cm below isocenter
2. Turn the ODI on

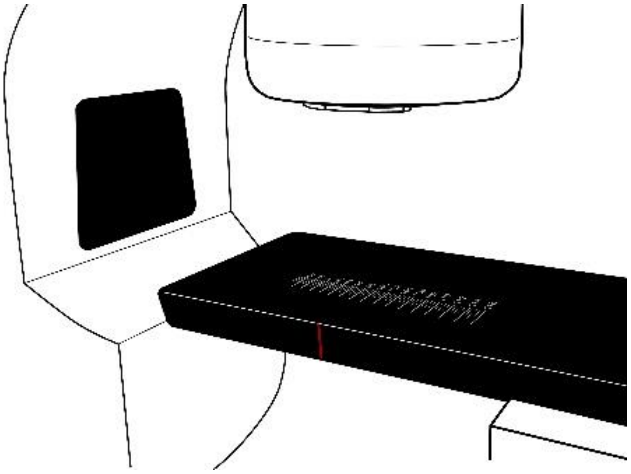

3. Position a block on the couch aligned with the crosshairs (see figure below), with a large surface facing the ODI hole.

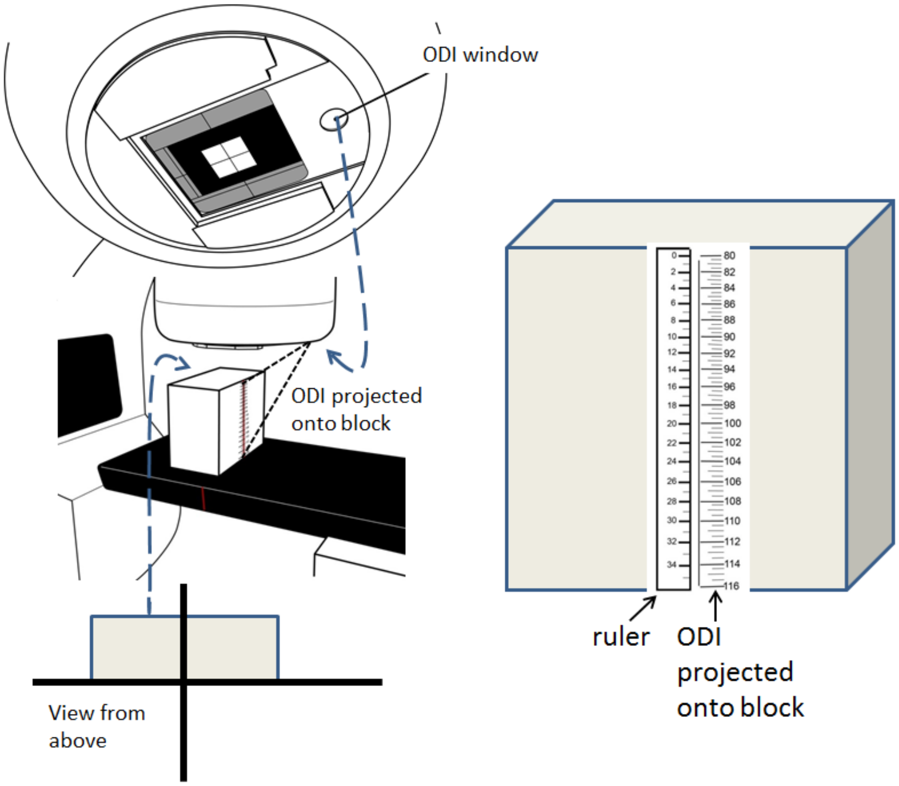

4. Check that the wall vertical lasers are just touching the front surface of the block.

5. Place a ruler on the vertical surface of the block. Turn the ODI on and measure the distance between the 80cm ODI mark and the 100cm ODI mark (should be 20cm).
4. Record the error in the measured distance.
5. Repeat this for the 90cm ODI and 110cm ODI (comparing both with the 100cm ODI mark).
6. Note: To cover this range, it may be necessary to move the couch vertically.

Preparation task: Accurately position the graph paper (later tasks refer to this).
Purpose: To accurately position graph paper for various mechanical tests

NOTE: this task must be completed before starting the next set of tests.

Procedure:

1. Set the gantry and couch to 0°, collimator to 0° *(hint: it can be useful to set the couch to 0° here – Test 15)*
2. Set the couch position to lateral = 0, vertical = 0, longitudinal = 140 (Varian) *(hint: this makes it easier for later tests)*
3. Adjust the couch vertical position so that the SSD is exactly 100cm to the surface of the graph paper (using mechanical distance indicator)
4. Remove the MDI and holder. Turn the field light on.
5. Position graph paper on the couch, aligned with the crosshairs.
6. Tape the graph paper in position.
7. Mark the isocenter point on the graph paper (in reference to the linac cross-hairs)

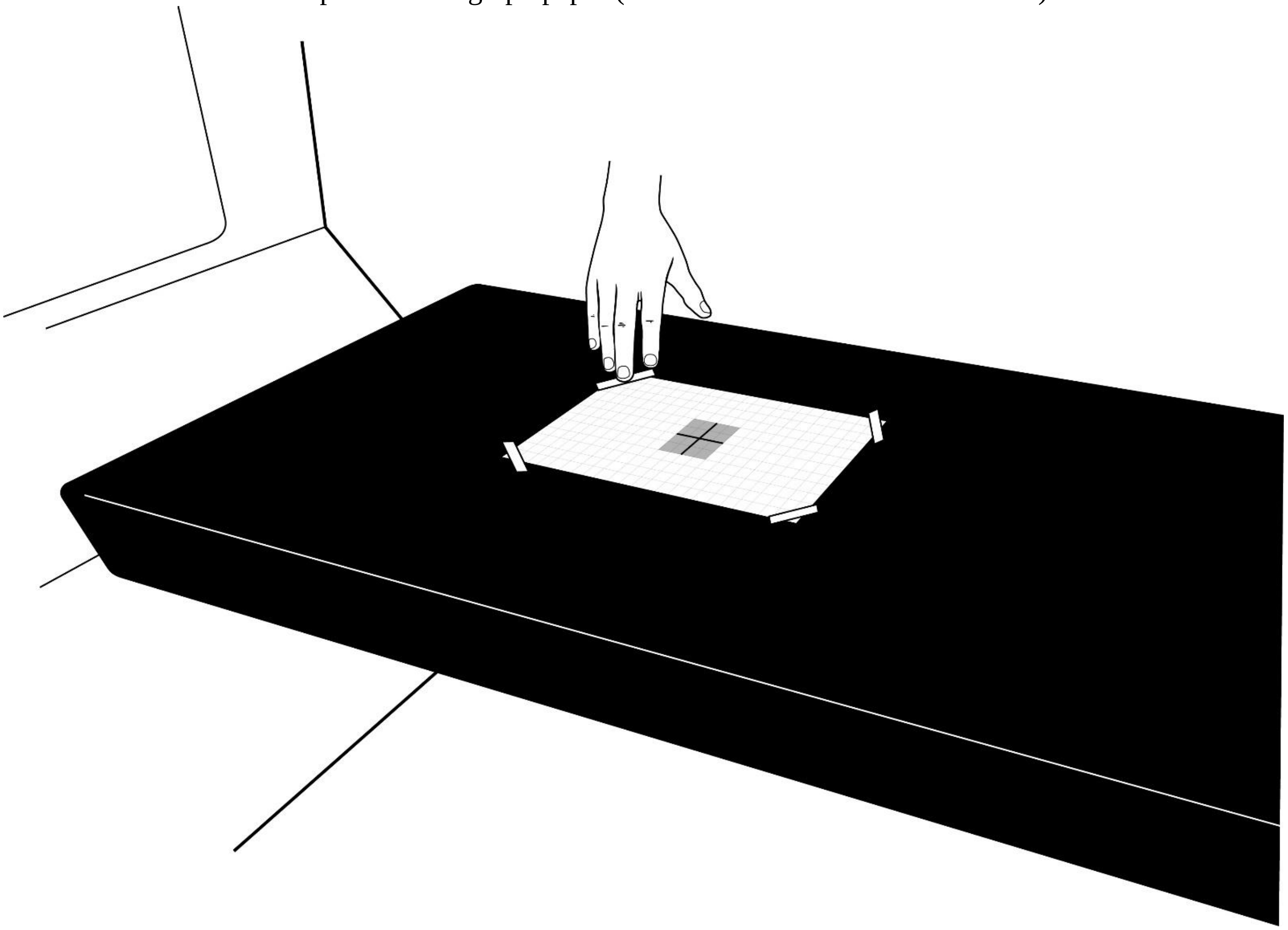

Test 7: Ceiling laser alignment at isocenter
Purpose: To test the accuracy of the lasers to define isocenter
Tolerance: 2 mm
Procedure to test ceiling laser alignment

1. [Set gantry to 0°, collimator to 0°, couch surface to 100SSD]
2. [Position graph paper on couch]
3. Rotate gantry until the ceiling laser is visible on the graph paper.

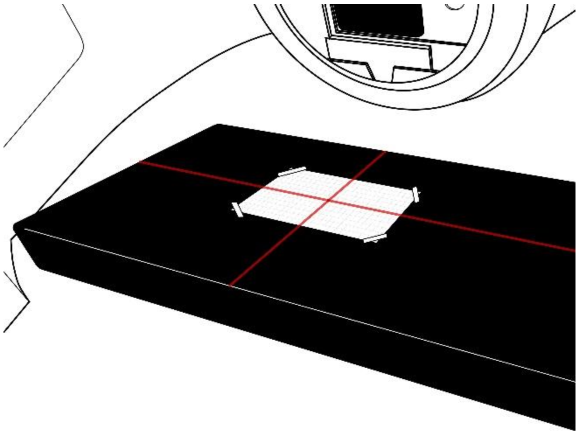

4. Check whether ceiling laser points to the isocenter as seen on the graph paper. Record the error in the laser position. *(hint: it is easy to confuse the ceiling and sagittal lasers – put your hand in front of the sagittal laser when doing this test)*

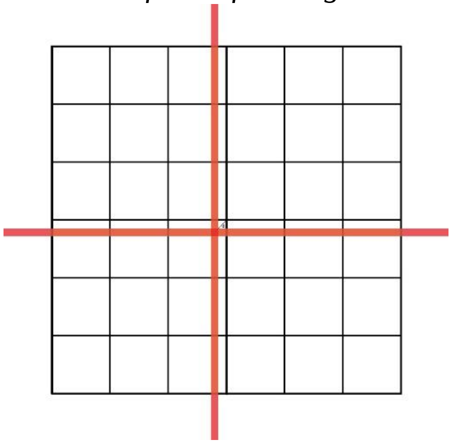

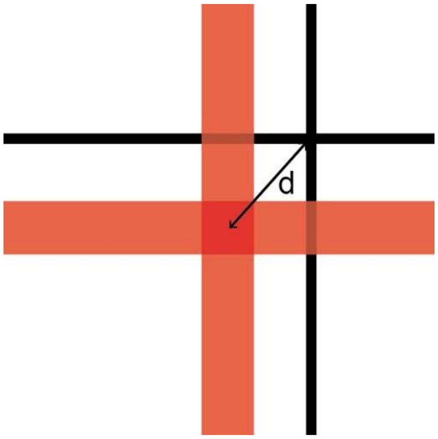

5. If there is a sagittal laser (at foot of couch), repeat this test with that laser

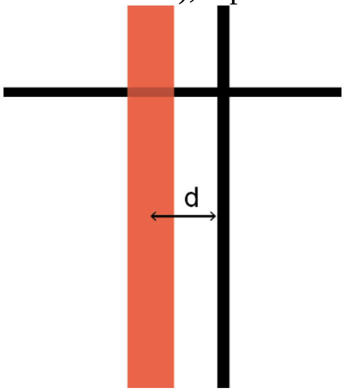

Test 8: Ceiling laser orientation
Purpose: to test the ceiling laser is oriented parallel to the couch motion
Tolerance: 2 mm

1. [Set gantry to 0°, collimator to 0°, couch surface to 100SSD]
2. [Position graph paper on couch]
3. Move couch to 30cm below isocenter (use the couch vertical readouts).

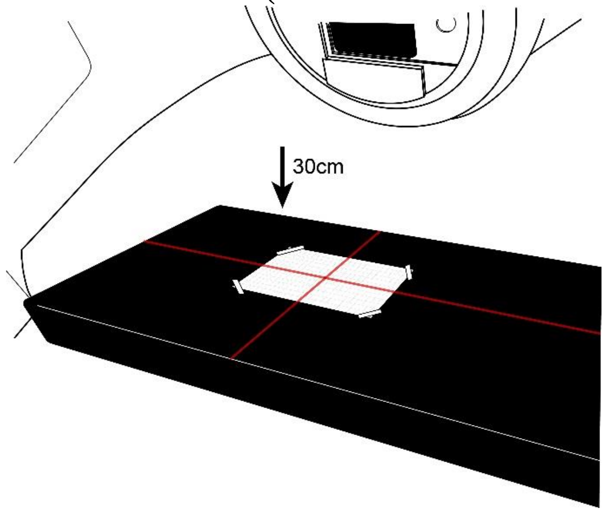

4. Check whether ceiling laser points to the isocenter on the graph paper (checks laser orientation). Record the error in the laser position.

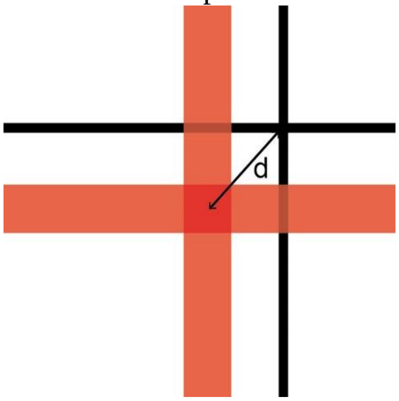

5. If there is a sagittal laser (at foot of couch), repeat this test with that laser

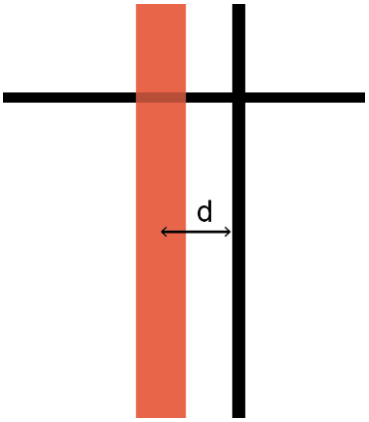

Test 9: Test vertical laser alignment (on side walls)
Purpose: To test vertical lasers (wall) are aligned with isocenter
Tolerance: 2 mm

1. Rotate the gantry to 0°.
2. Set 100cm SSD to the top of the graph paper
3. [Position graph paper on couch]
4. Hold paper in front of isocenter and check that the vertical (wall) lasers (both sides) are aligned with isocenter (as seen on the graph paper)

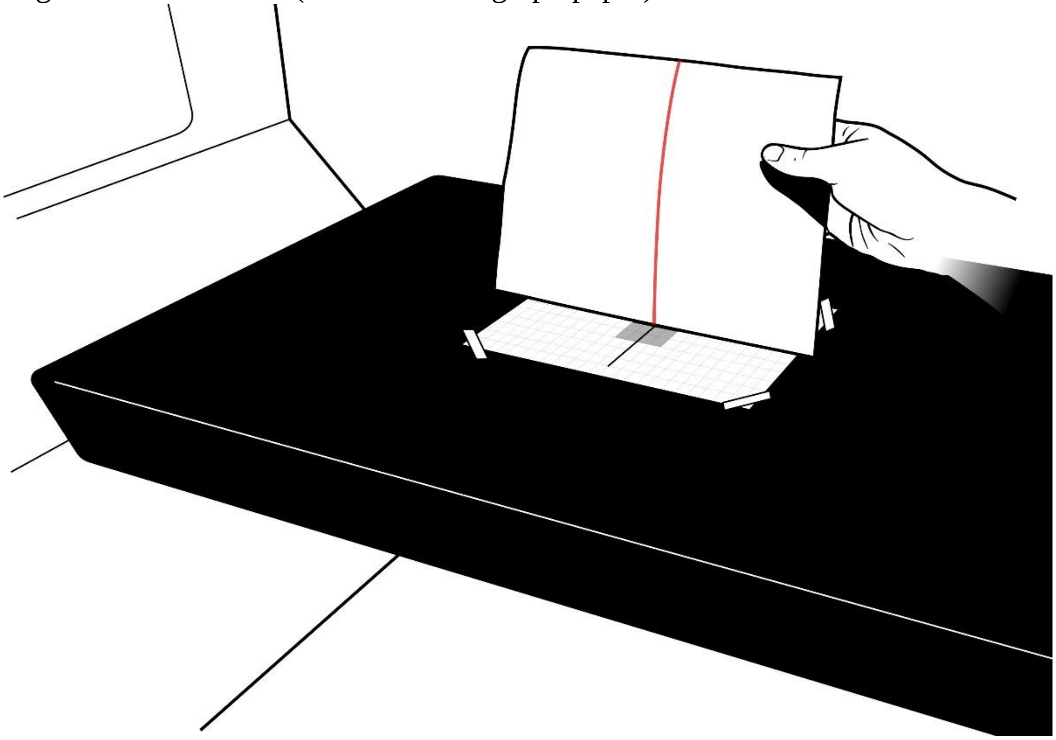

5. Record the error in the vertical laser position for both wall vertical lasers.

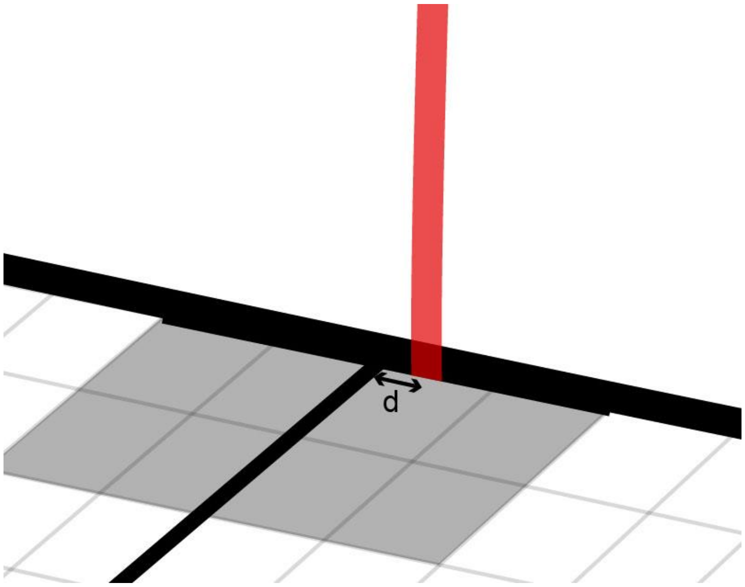

Test 10: Cross-hair centering
Purpose: To determine the integrity of the cross-hair under collimator rotation
Tolerance: 1.0 mm
Procedure:

1. [Set the gantry, collimator, and couch to 0°]
2. [Position graph paper on the couch]
3. Set jaws to the largest field possible (e.g. 40x40cm^2)
4. Turn the field light on, and leave on for the remainder of this test.
5. Rotate the collimator through the full range

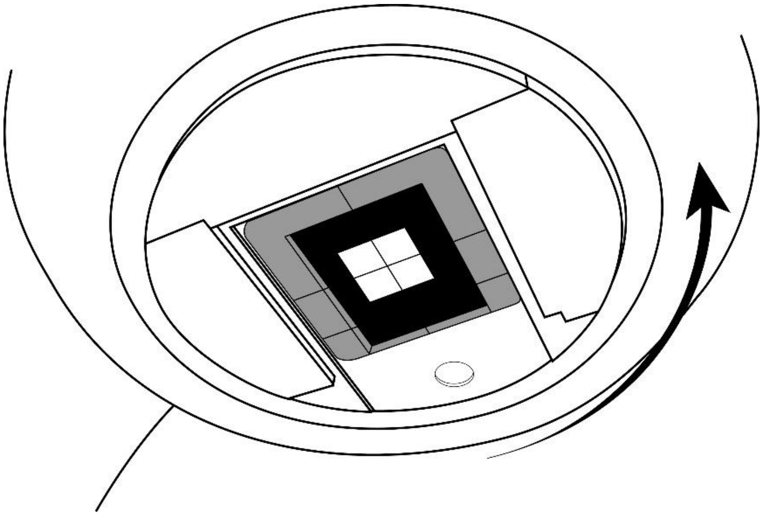

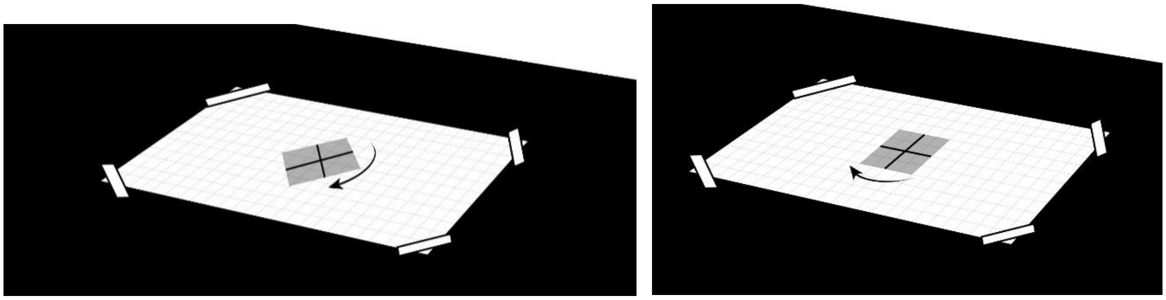

6. For each 90° rotation, note the distance between position of the crosshair and the original isocenter mark on the graph paper, and record the maximum deviation (checks centering)

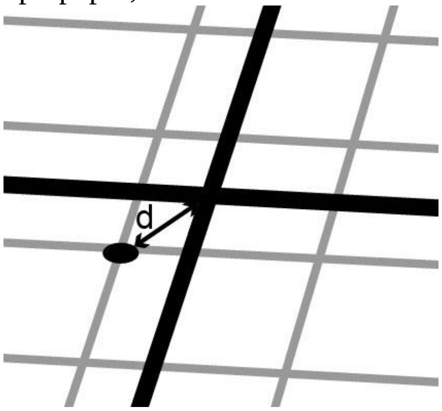

Test 11: Accuracy of asymmetric jaw readouts (compared with light field)
Purpose: To test the accuracy of the electronic readout against actual field
Tolerance: 1 mm
Procedure:

1. [Tape graph paper to the couch at isocenter (100cm SSD, using the mechanical distance indicator)]
2. [Set the gantry, collimator, and couch to 0°]
3. Start with the largest possible field (e.g. 40x40cm)
4. Set the jaws to different positions (as listed in the results sheet) by comparing the light field on the graph paper.
5. One-by-one, fine-tune each jaw position by carefully moving the jaw until it matches the graph paper (i.e. using the light field, <u>not</u> the digital readouts).
6. Record the digital readout for the jaw you are testing
7. Repeat this for all 4 jaws: X1, X2, Y1, Y2
8. When testing the 0cm position for each jaw, also compare the crosshair and jaw position along the length of the jaw. This is a check that the crosshair is parallel to the jaws.

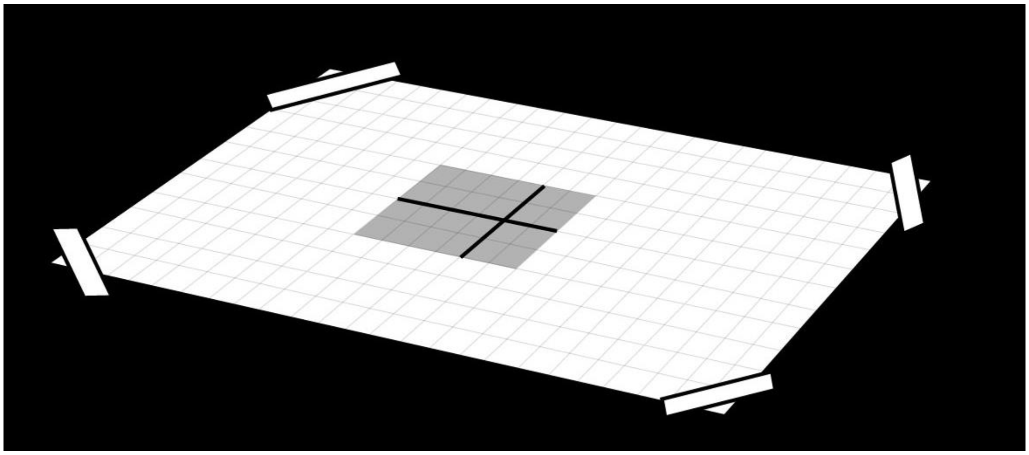

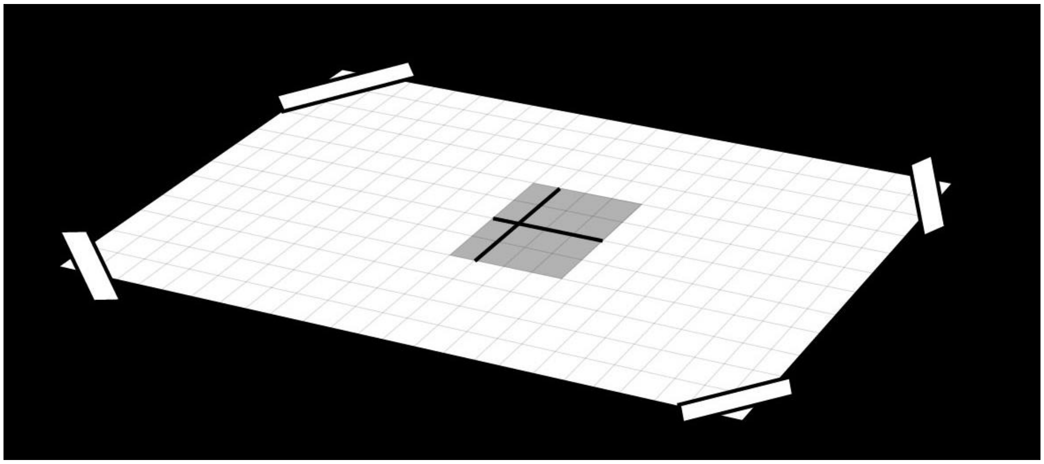

(hint: if the graph paper isn't large enough in one dimension, try rotating the collimator by 90°, or shifting the couch – just remember to put back!)

Test 12: MLC position vs. light field
Purpose: To check that the MLCs are moving to the correct positions
Tolerance: 2 mm
Procedure:

1. [Set the gantry and collimator to 0°]
2. [Set the couch to isocenter]
3. [Tape graph paper to the couch at isocenter (100cm SSD)]
4. Load the MLC pattern file
    - e.g. Folder: Program files/Varian/Oncology/MLC/Plans/ATP Plans/MLC ATP Ver 3/Mill120/ , File: ATP, Millennium120(statis) StatML20.mlc, Sequence: Leafposition15cm, and others
5. Compare expected light field and actual light field (of the MLC shape) using graph paper taped to the couch. Record the difference between the set positions (as indicated on the linac monitor) and actual positions (as seen on the graph paper).
6. Repeat for several MLC patterns.

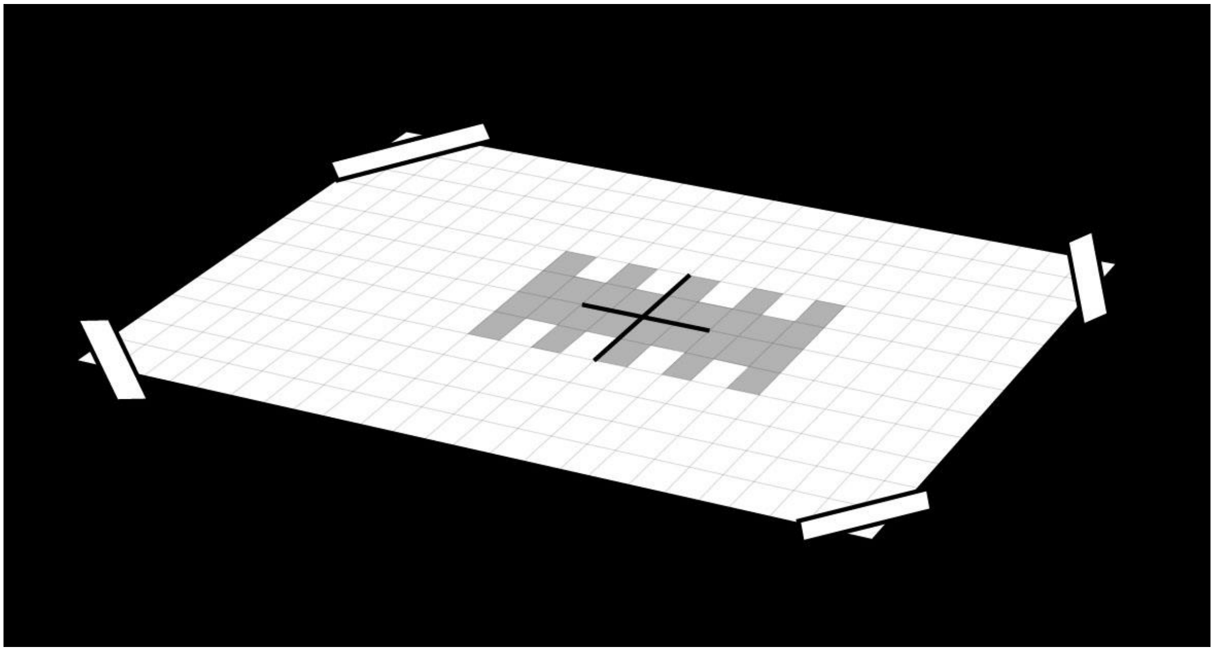

Test 13: Accessory positioning check
Test 13A: Physical Wedge Position Accuracy
Purpose: To check the integrity of the wedges (i.e. that it hasn't shifted position in its housing)
Tolerance: 2 mm
Procedure:

1. [Set the gantry and collimator to 0°, position graph paper]
2. Insert each wedge into the accessory slot, heel in.

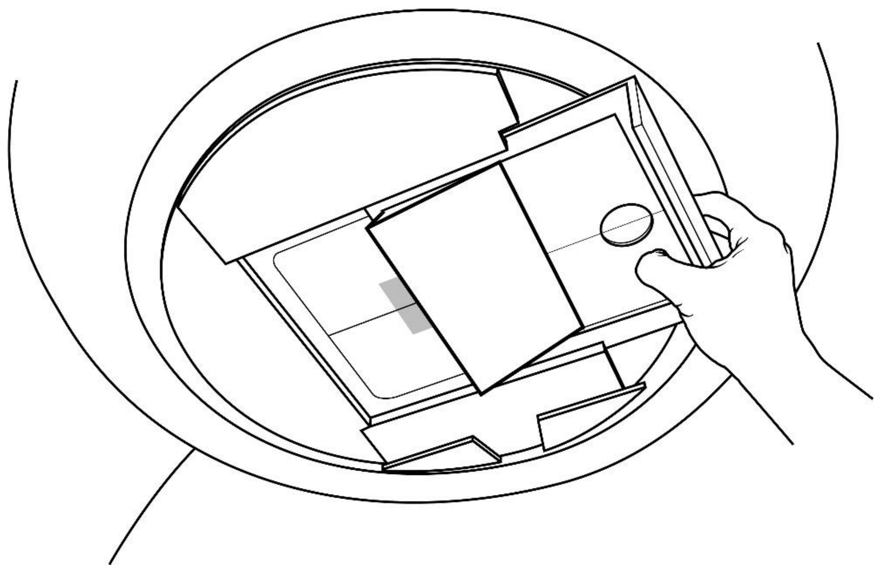

3. Set the jaws to the maximum field size (e.g. 40x40cm^2)
4. Measure distance from isocenter (identified on the graph paper) to the edge of the shadow on the toe side of the wedge. Record this distance.

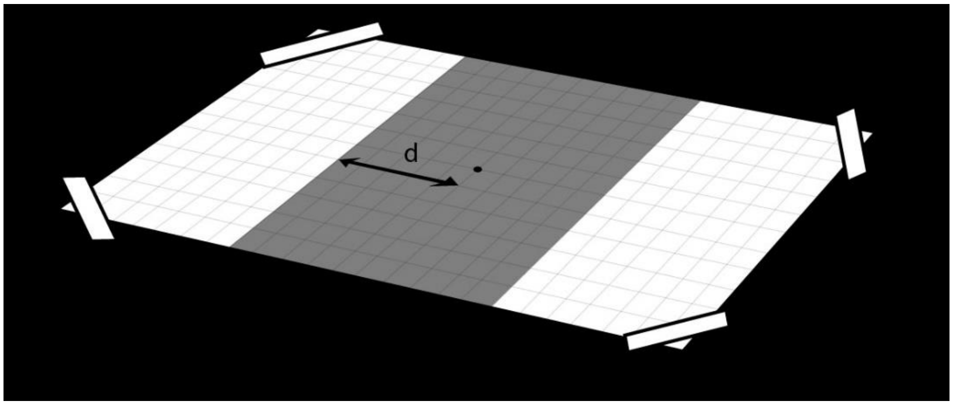

5. Repeat for each wedge.
6. Repeat for lower wedges (if used clinically)

Note: The baseline values for the wedge dimensions must be established at the time that the wedge factor is measured.

Test 13B: Physical graticule position check

Purpose: To check the integrity of the graticule (i.e. to check that the physical graticule is aligned with the crosshairs)

Tolerance: 2 mm

Procedure:

1. [Set the gantry and collimator to 0°, position graph paper]
2. Open the jaws to the maximum field size.
3. Insert the physical graticule

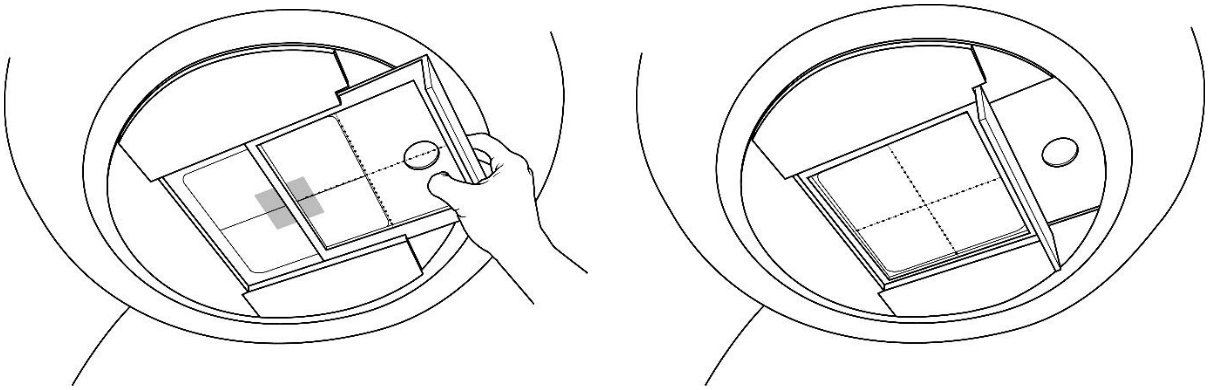

4. Check that the cross hairs and graticule shadow are in agreement
5. Record the difference between the cross hairs and the graticule shadow.

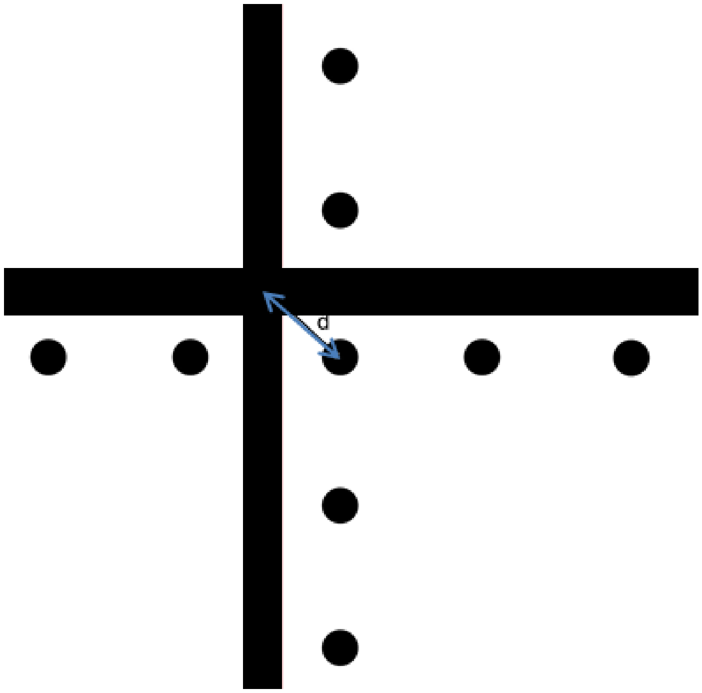

Test 14: Couch angle vs. readout <u>and</u> couch centering (TWO tests)
Purpose: To test the accuracy of the couch angle readout and the accuracy of the couch centering
Tolerance: 1° (electronic readout), 1° (mechanical readout), 1 mm (radius of couch centering)
Procedure:

1. [Set the gantry and collimator to exactly 0° using the Level]
2. Put some blank paper on the couch, and mark isocenter using the cross hairs (do NOT remove the graph paper – it's still needed)
3. Check the couch 0° position:
    a. Move the couch in and out ±20cm. The couch is at 0° if the isocenter marked on the graph paper tracks the longitudinal cross hair.
    b. If this is not the case, find the couch 0° position as follows:
        i. Rotate the couch until it moves in line with the crosshairs. This is the true couch 0°.
        ii. Remove the blank paper. If the graph paper is no longer aligned with the crosshairs, reposition the graph paper (as described earlier).
4. Record the digital couch angle readout

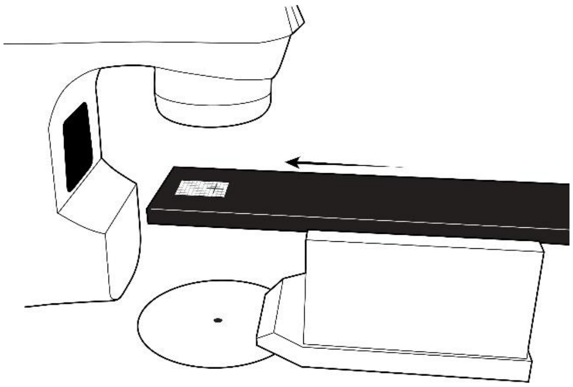

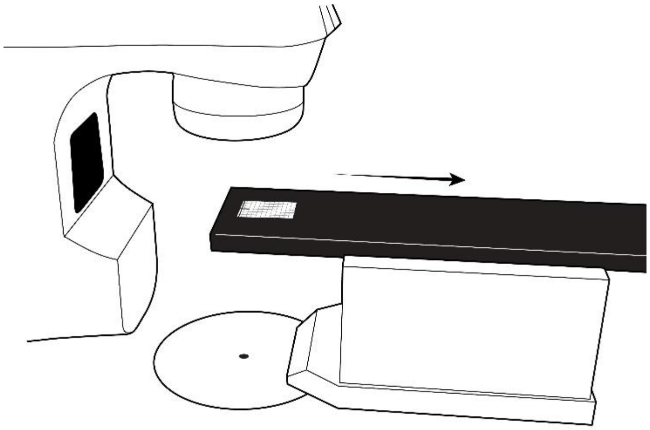

5. Rotate the couch to approximately 270°.

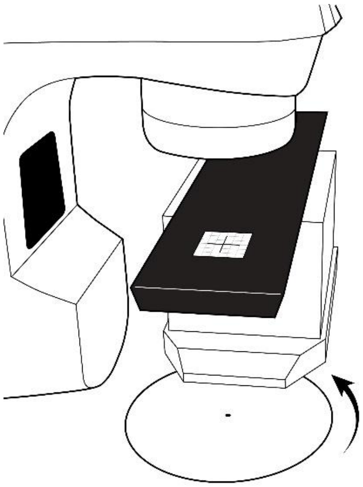

6. Fine-tune the couch rotation until the lines on the graph paper are parallel to the linac crosshairs.

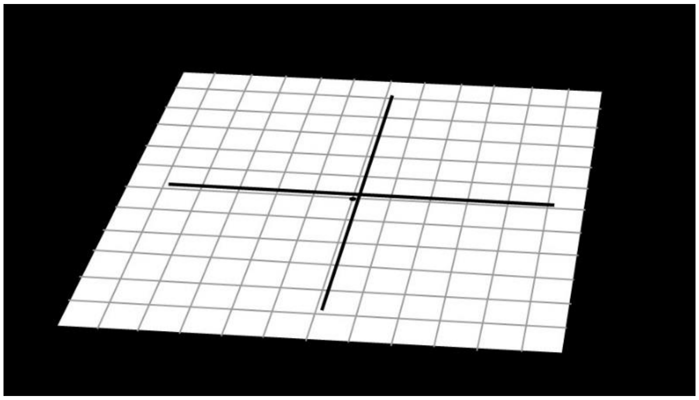

7. Record the digital readout (and mechanical readout if used) (checks readout).
8. Check the distance between position of the crosshair and the original isocenter mark on the graph paper (checks couch centering). Note this distance, and record the maximum deviation that you see when rotating the couch.

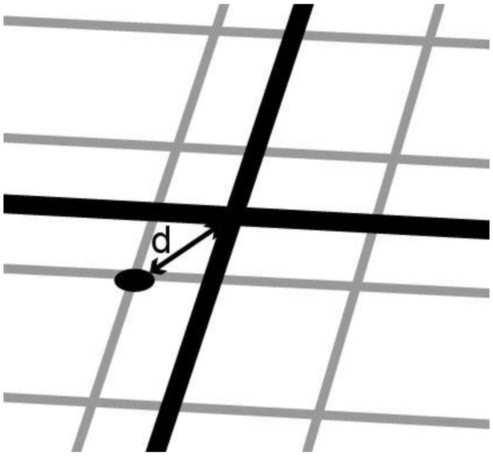

9. Rotate the couch to approximately 90° and repeat steps 6-8.

Test 15: Relative couch indexing tests
Purpose: To test the accuracy of absolute and relative couch shifts using the readouts
Tolerance: 2 mm

Test 15A: Procedure to test relative lateral couch positions (‘couch lat’)
Tolerance: 2 mm

1. Set gantry, collimator and couch to 0°, and couch lateral position to 0.
2. [Tape graph paper to couch top, centered at the crosshairs]
3. Read the couch lateral position from the digital readouts
4. Shift the couch left 10cm using the crosshair position on the graph paper.

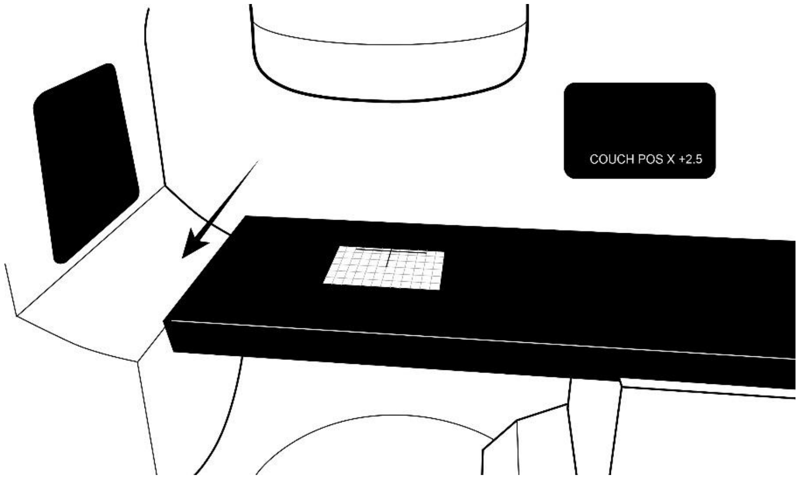

5. Read the couch lateral digital readout, and record the couch lateral coordinates (should be -10cm – if you started at 0, then the readout should be 990 (Varian)).
6. Shift the couch back to isocenter (using the crosshairs)
7. Shift the couch right 10cm using the crosshair position on the graph paper

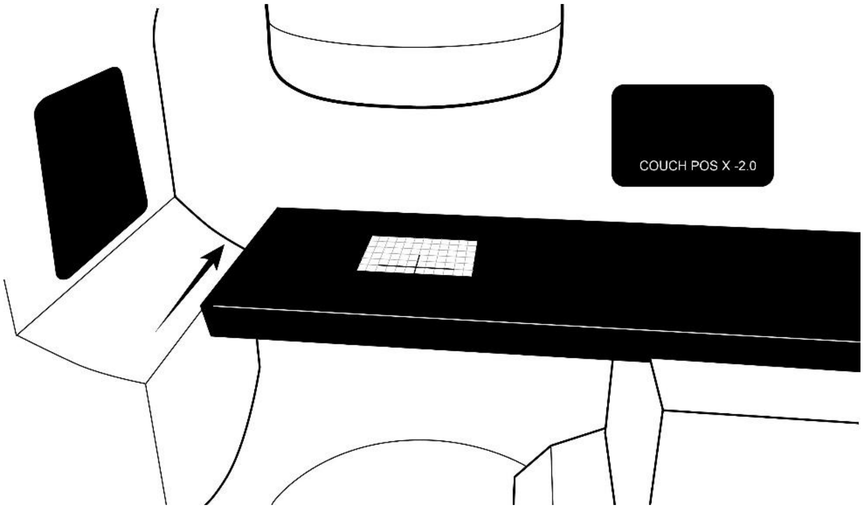

8. Read the couch lateral digital readout, and record the couch lateral coordinates (should be +10cm – if you started at 0, then the readout should be 10 (Varian)).

<u>Test 15B</u>: Procedure to test relative longitudinal couch positions ('couch lng')
Tolerance: 2 mm

1. Shift the couch back to isocenter (aligning graph paper and crosshairs)
2. Read the couch longitudinal position from the digital readouts *(hint: easiest if set to140 for a Varian unit)*
3. Shift the couch <u>in</u> 10cm using the crosshair position on the graph paper.

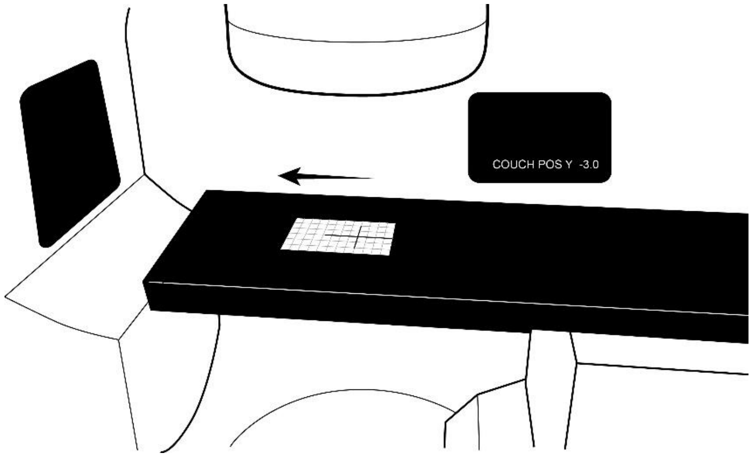

4. Read the couch digital longitudinal readout, and record the <u>change</u> in coordinates (should be +10cm – if you started at 140, then the readout should be 150 (Varian)).
5. Shift the couch back to isocenter
6. Shift the couch <u>out</u> 10cm using the crosshair position on the graph paper.

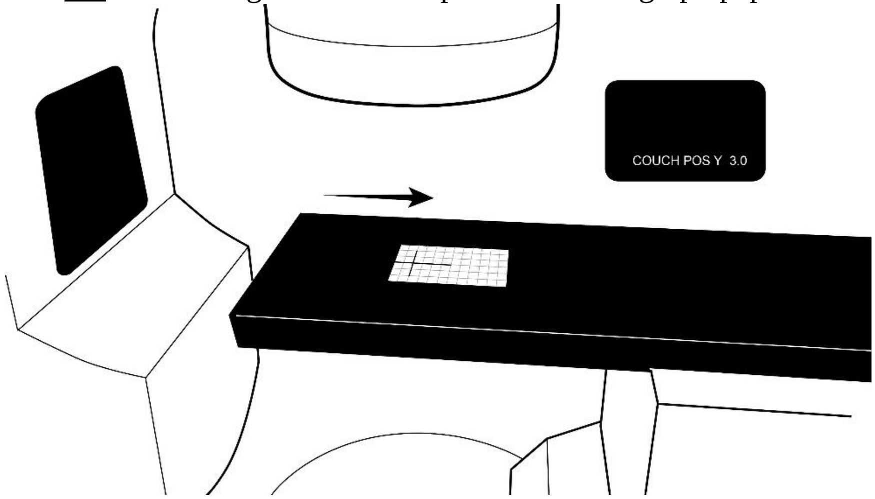

7. Read the couch longitudinal readout, and record the <u>change</u> in coordinates (should be -10cm – if you started at 140, then the readout should be 130 (Varian)).

<u>Test 15C</u>: Procedure to test relative vertical couch positions ('couch vert')
Tolerance: 2 mm

1. Re-center the couch by aligning the graph paper (by moving couch) and the crosshairs.
2. Read the couch vertical digital readout *(hint: easiest if set to 0)*
3. Decrease the couch height by 10.0cm using the optical distance indicator (ODI) (set ODI=110cm)
4. Record the digital couch vertical position
5. Repeat steps 3 and 4 for 20.0 below isocenter.

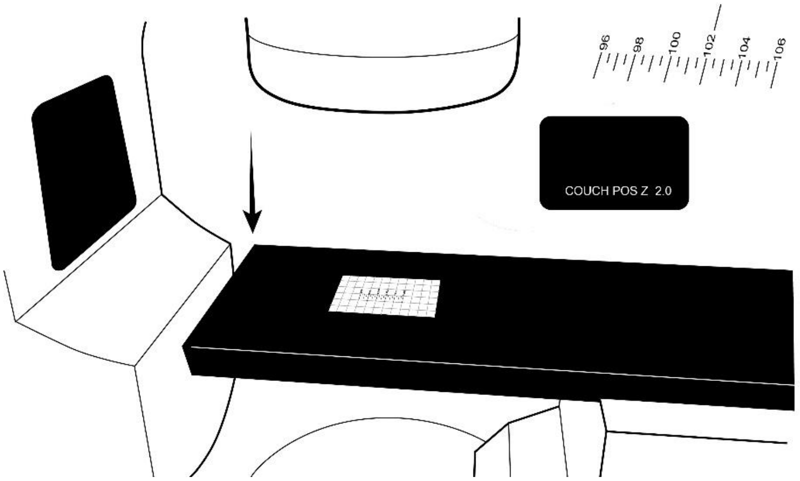

Test 16: Absolute couch indexing tests
Procedure to test absolute position of couch (do at end, as need to remove graph paper):
Tolerance: 2 mm

1. Set gantry to 0°.
2. Set couch height to 100cm SSD using mechanical distance indicator (MDI). Remove the MDI.
3. Attach Exact couch calibration bar to 0 position on couch
4. Move the couch laterally and longitudinally until the cross on the calibration bar aligns with the linac crosshairs.
5. Record the digital couch readouts. These should read 0cm (lat), 0cm (vert) and 140cm (long) (Varian values).

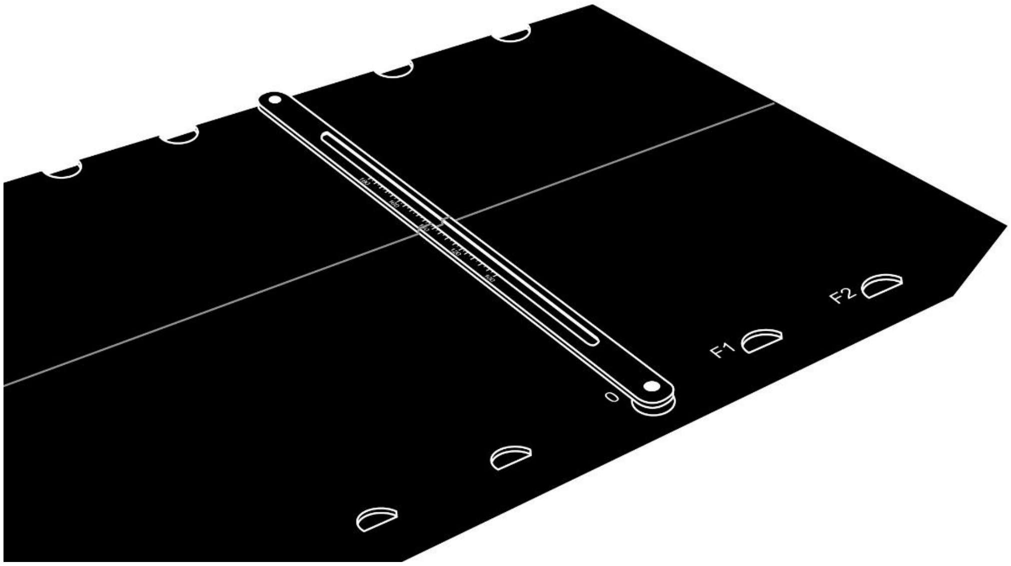

Test 17: Accessory position check
Purpose: To check that the accessories fit in the holder accurately and safely
Tolerance: Functional
Procedure:

1. Visual check that accessories insert and latch correctly for each accessory slot, and are not excessively loose.
2. Record whether the accessory fits snuggly into the accessory slot.

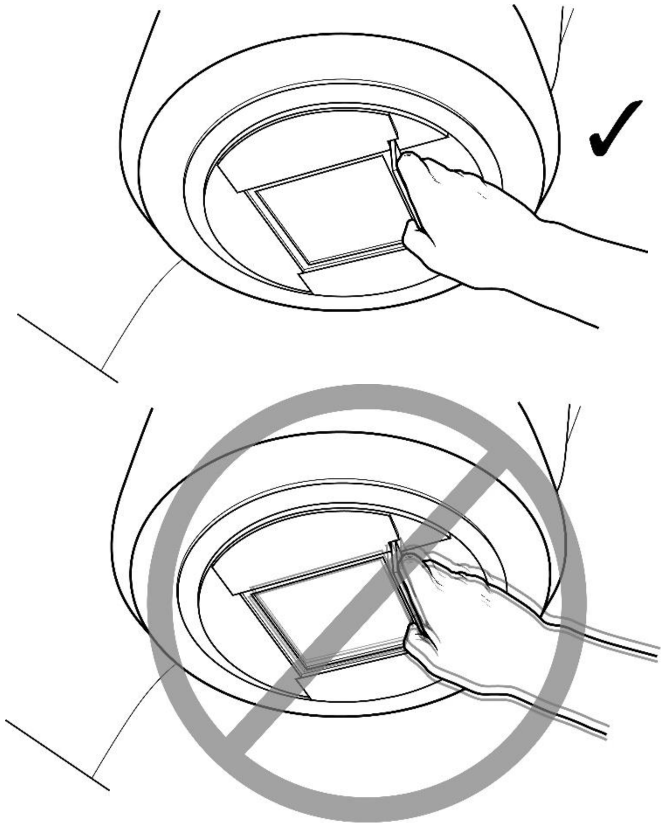

3. Repeat for each accessory (each electron applicator, etc.) *(hint: this test can be combined with 13A and 13B for physical wedges and graticule)*

Test 18: Safety tests

Procedure:

A. Door interlock: Run beam. Open the door, and check that the beam is turned off.
B. Warning lights (in-room and outside door): Run beam. Check that each light is operating. For lights inside the room, use the video monitors.
C. Backup counter: Run beam for 100MU, and check that the backup counter reads 100MU. For Varian Clinac machines, the backup counter is located on the inside of the electronics cabinet. For Truebeam machines, the backup counter is located on the console.
D. Pendant:
    a. Visually inspect each pendant (couch and EPID) for damage, loose screws etc.
E. Integrity of accessories:
    a. Visually inspect each wedge (8 wedges) and cone (5 cones) for damage, loose screws etc.
    b. For each cone, check that the insert pin moves and that the latch works.
F. Examine the couch for damage/hazards (sharp edges etc.)
G. Check the collision interlocks on the imaging detector and arm (if present) are operational
H. Beam off interlock: Run beam. Push “Beam-off” at the linac console, and check that the beam turns off.
I. Check the Audio-Visual monitors are functioning properly

Monthly LINAC QA tests
Date: Signature:

NOTE: The acceptable ranges are from AAPM TG-142, table II, for non-IMRT machines. Users should verify and edit these values according to their own environment. Also, it is important to note that the inter-dependence of various parameters being tested here means that the cause of the failure is not necessarily obvious, and well-trained staff are still needed to determine the cause of any failures.

test	Parameter	positions	Recorded value	Acceptable range	Pass/Fail	Comments
Test 1	Gantry angle vs. readout	0°		359.0 – 1.0°		
		90°		89.0 – 91.0°		
		180°		179.0 – 181.0°		
		270°		269.0 – 271.0°		
		180°		179.0 – 181.0°		
Test 2	Collimator angle vs. readout	0°		359.0 – 1.0°		
		90°		89.0 – 91.0°		
		270°		269.0 – 271.0°		
Test 3	Left wall laser orientation	(linac exit window)		≤2mm		
	Right wall laser orientation	(linac exit window)		≤2mm		
Test 4	Left wall laser alignment (horizontal laser)	Isocenter		≤2mm		
	Right wall laser alignment (horizontal laser)	Isocenter		≤2mm		
Test 5	ODI @ 100cm	100.0cm		99.8 – 100.2cm		
Test 6	ODI (other distances, relative to 100cm point)	80 – 100cm		19.8 – 20.2cm		
		90 – 100cm		9.8 – 10.2cm		
		110 – 100cm		9.8 – 10.2cm		
Test 7	Ceiling laser alignment	Isocenter		≤2mm		
	Sagittal laser alignment (if present)	Isocenter		≤2mm		
Test 8	Ceiling laser orientation	Isocenter		≤2mm		
	Sagittal laser orientation (if present)	Isocenter		≤2mm		
Test 9	Left wall laser alignment (vertical laser)	Isocenter		≤2mm		
	Right wall laser alignment (vertical laser)	Isocenter		≤2mm		
Test 10	Cross-hair centering	Full rotation of collimator		≤1mm		
Test 11	Jaw readouts vs. light field (Y1 jaw, asymmetric)	-10cm		-9.9 – -10.1 cm		
		0 cm		-0.1 – 0.1 cm		
		10 cm		9.9 – 10.1 cm		
		20 cm		19.9 – 20.1 cm		

	Jaw readouts vs. light field (Y2 jaw, asymmetric)	-10 cm		-9.9 – -10.1 cm		
		0 cm		-0.1 – 0.1 cm		
		10 cm		9.9 – 10.1 cm		
		20 cm		19.9 – 20.1 cm		
	Jaw readouts vs. light field (X1 jaws, asymmetric)	-2 cm		-1.9 – -2.1 cm		
		0 cm		-0.1 – 0.1 cm		
		10 cm		9.9 – 10.1 cm		
		20 cm		19.9 – 20.1 cm		
	Jaw readouts vs. light field (X2 jaw, asymmetric)	-2 cm		-1.9 – -2.1 cm		
		0 cm		-0.1 – 0.1 cm		
		10 cm		9.9 – 10.1 cm		
		20 cm		19.9 – 20.1 cm		
	Crosshair parallel to x jaw					Parallel to jaw along entire length
	Crosshair parallel to y jaw					Parallel to jaw along entire length
Test 12	MLC pattern check (using light field)	(MLC patterns)		≤2mm		
Test 13A	Physical wedge position check	Wedge 1 (15° upper wedge)				2mm tolerance – need reference values
		Wedge 2 (30° upper wedge)				2mm tolerance – need reference values
		Wedge 3 (45° upper wedge)				2mm tolerance – need reference values
		Wedge 4 (60° upper wedge)		8.1 – 8.5cm		(these are reference values for our wedges)
		(add lower wedges, if used)				2mm tolerance – need reference values
Test 13B	Physical graticule position			≤2mm		
Test 14	Couch angle vs readout	0°		359.0 – 1.0°		
		90°		89.0 – 91.0°		
		270°		269.0 – 271.0°		
	Couch centering	Full couch rotation		≤1mm		
Test 15A	Couch relative position (lateral)	-10.0 cm (990.0*)		989.8 – 990.2 cm		
		+10.0 cm (10.0*)		9.8 – 10.2 cm		

Test 15B	Couch relative position (longitudinal)	-10.0 cm (130*)		129.8 – 130.2 cm		
		+10.0 cm(150*)		149.8 – 150.2 cm		
Test 15C	Couch relative position (vertical)	0 cm		≤2mm		
		10 cm		≤2mm		
		20 cm		≤2mm		
Test 16	Couch absolute position (isocenter)	Lateral		≤2mm		
		Longitudinal		≤2mm		
		vertical		≤2mm		
Test 17	Accessory position check	All accessories		Tight fit		
Test 18A	Door interlock			Functioning		
Test 18B	Warning lights	Light 1 (in room)		Operational		
		Light 2 (in room, if applicable)				
		Light 3 (in room, if applicable)				
		Outside treatment vault door				
Test 18C	Backup counter	100MU		Correct		
Test 18D	Pendants	Pendant 1		Operational		
		Pendant 2 (if applicable)				
		Pendant 3 (if applicable)				
Test 18E	Integrity of accessories			Acceptable		
Test 18F	Integrity of couch			Acceptable		
Test 18G	Collision interlocks (if applicable)	Check all installed collision interlocks		Functioning		
Test 18H	Beam off interlock			Functioning		
Test 18I	Audi-visual monitors			Functioning		

Note: values marked with a ‘*’ are for Varian linacs.
